# It takes two: examining the dynamic nature of cooperative behavior in adolescents

**DOI:** 10.3389/fpsyg.2024.1269016

**Published:** 2024-04-29

**Authors:** Taryn Berman, Isabelle Plante, Mathieu Roy

**Affiliations:** ^1^Department of Psychology, McGill University, Montreal, QC, Canada; ^2^Department of Didactics, Université du Québec à Montréal, Montreal, QC, Canada

**Keywords:** trust, cooperation, cooperative behavior, adolescence, social skills, impulsivity

## Abstract

Cooperating with those around us is an important facet of functioning in modern-day society. Forming successful cooperative relationships requires trust, reciprocity, and other interpersonal skills that continue to develop during adolescence. This study examined the dynamic nature of how trust is formed and broken among 248 adolescents (Males = 110, *M*_Age_ = 15.1 years) throughout an iterative cooperative task (i.e., the Trust Game) and the interindividual differences that alter the success of their relationships. In our study, adolescents from the same classroom were anonymously paired and played a 10-trial version of the Trust Game, which examines trust and reciprocity. We found that trust is formed in the first half of the game and decreases as the threat of defection nears in the last trial. As the game progressed, the relationship between trial number and investments on the subsequent trial was mediated by percent return (*ab* = −0.09, 95% CI = [−0.15, −0.02]). Importantly, this relationship was moderated by social skills (*p* = 0.003) and impulsivity (*p* = 0.001), such that increases in either were associated with decreased percent return and investments on future trials. Overall, we found that cooperation is an adaptive behavior which requires trust and reciprocity, and adolescents need to exhibit both of these behaviors to have fruitful interactions. These findings suggest that interventions to help students think about their partner’s perspective and stress the longer-term nature of interactions with peers would foster successful cooperation in social situations.

## 1 Introduction

Cooperation is crucial in forming the trusting and mutually beneficial relationships that are at the heart of our society. Importantly, cooperation is a dynamic process involving trust and reciprocation among multiple individuals. Many of the skills required to cooperate develop during adolescence. More specifically, during adolescence individuals’ behavior transitions from being self-oriented (i.e., concerned with how situations benefit themselves) to other-oriented (i.e., concerned with how the situation benefits others), where they develop the skills required to maintain increasingly complex social relationships (Eisenberg et al., 1995; Martin et al., 2008; Crone and Dahl, 2012). Adolescence is also a period where heightened attention is paid to peers and individuals learn from interpersonal conflict with said peers (Martin et al., 2008; Crone and Dahl, 2012). Social problems that could be associated with – or lead to – poor cooperation begin to emerge in late childhood and early adolescence, but become less prominent in adulthood (Crone and Dahl, 2012). For this reason, it is important to study cooperative behaviors while they are still developing in adolescents, a turning point for acquiring such skills.

Cooperation is both necessary and difficult to do because it requires trust (van de Groep et al., 2020). Although challenging, learning to trust is established through reciprocation, despite the ever-present temptation against reciprocation and toward personal short-term gain (van de Groep et al., 2020). Furthermore, measuring both trust and reciprocation is challenging, but essential to capture the dynamic nature of cooperation. To do so, the current study included dyads of adolescents interacting in a cooperative task, the Trust Game. Contrary to previous work on the topic – which typically involved individuals playing against a computer – the current study included two actual adolescent players from the same classroom who were paired anonymously. This allows us to differentiate the individual contribution of each player, and the ability for each player to adapt to the other player’s behavior in order to promote cooperation within the dyad. This paper also examines the influence of interindividual differences on the quality of cooperation. This knowledge will allow us to predict who will struggle to cooperate and why, making it possible to improve their cooperative skills.

Despite the complexity of cooperative interactions, prior research has shown that basic cooperative skills begin to develop in children as young as 3 years old (Olson and Spelke, 2008). These behaviors continue to mature during adolescence, a time where self-control, social inhibition, and empathy are rapidly developing (van de Groep et al., 2020; Napolitano et al., 2021). As such, adolescents display less reciprocity and cooperative behaviors compared to adults (Belli et al., 2012). Perspective-taking – a pro-social behavior where an individual perceives a situation from the point of view of another person – continues to develop during adolescence and is associated with better and more cooperative social interactions (Eisenberg et al., 1995; Martin et al., 2008; Fett et al., 2014). Notably, the interdependent nature of cooperation makes it challenging to examine and requires studying all individuals involved in the interaction. Research has shown that it takes decades to develop the social abilities necessary for optimal cooperation (van de Groep et al., 2020) – although this development occurs mainly during adolescence – and lack of cooperation during adolescence can lead to the re-evaluation of social relationships based on the behavior of others (Schilke et al., 2013).

Typically, research examining cooperative behaviors employed self-report measures to assess how cooperation may be related to personality traits and skills (Glaeser et al., 2000; Evans and Revelle, 2008; Alós-Ferrer and Farolfi, 2019). The goal of these questionnaires was to develop an inventory analogous to cooperative tasks examining trust, with items ranging in number from one to nearly 100 (Alós-Ferrer and Farolfi, 2019). When compared to tasks, the questionnaires yielded conflicting results regarding their efficacy (Glaeser et al., 2000; Evans and Revelle, 2008; Ben-Ner and Halldorsson, 2010). Studies have shown that individuals are unable to predict their future behavior and accurately justify their responses (Nisbett and Wilson, 1977), suggesting that people may not have good insight into how well they cooperate. In general, self-report questionnaires may be biased due to poor introspection abilities (i.e., poor discernment into how well you cooperate). Importantly, people want to be good at cooperating with others; however, the issue with being unable to determine your own cooperative abilities is that people may think they cooperate well, when in reality they do not. Notably, questionnaire data lack the richness of interaction present in dyads – as they only consider the perspective of one individual – displaying the importance for assessing cooperation in a fashion more similar to real-life situations.

In order to examine cooperation in a context with real interactions, economic games have been constructed to simulate cooperative social interactions involving social exchange. One of such tasks is known as the Trust Game (Berg et al., 1995), which is used due to its ability to be both dynamic and iterative. In this task, individuals are anonymously paired and have the goal of each earning as much money as possible. The *investor* gives money to the *trustee* and the amount is tripled prior to being received by the trustee. The trustee then decides how much of their earnings to share back with the investor. In an iterative version of this task, the pair plays multiple rounds of the game which allows for a more complex examination of the relationship formed, including failure to cooperate within a pair (King-Casas et al., 2005). The behavior of each individual player in this game provides important information regarding their success or failure in forming a trusting and cooperative relationship. The amount of money sent by the investor is a measure of trust in the trustee, and the sum returned reflects reciprocity and trustworthiness (Berg et al., 1995; Alós-Ferrer and Farolfi, 2019).

For cooperation and trust to be maintained, the trustee must return at least the investor’s initial investment. If this does not occur, trust has been ruptured and may never be regained (King-Casas et al., 2008). Ruptured trust can lead to less money earned overall for the trustee and is indicative of a less cooperative interaction. Using a fixed-choice modified version of the Trust Game, Ibáñez et al. (2016) found that higher levels of trustee impulsivity were associated with less returns. In line with the impulsivity literature, this suggests that impulsive trustees are unable to resist the temptation of immediate higher rewards, making it more difficult to maintain long-term cooperative relationships.

Previous studies have used economic games – like the Trust Game - to study cooperation as it unfolds in real-time, bypassing the problems inherent to questionnaires. When used with adolescent participants, most studies had participants – sometimes unknowingly – interact with a computer in lieu of another person (van den Bos et al., 2010; Lee et al., 2016; van de Groep et al., 2020). The Trust Game was used to show that reputation of the partner (Lee et al., 2016), empathy, risk, gender, and age (van de Groep et al., 2020) all influence behavior in the game. Imperatively, none of these studies assessed how cooperation develops through interactions between two real-players in an iterative fashion. Solely examining one side of the interaction only provides half of the information and vastly oversimplifies the story, in order to study a dynamic interaction both members of said relationship need to be examined.

The goal of cooperative tasks – like the Trust Game – is to gain trust, despite backward induction. In the Trust Game, players must build trust, while knowing that trust will likely be broken by their partner at the end of the game (Evans and Krueger, 2011). We propose two different extremes for explaining the behavioral continuum in this task: rational vs. optimal behaviors. According to backward induction, cooperation should never occur in this task, because both players know the rational strategy for the player who makes the last move (i.e., to keep everything; Evans and Krueger, 2011). From that position, it is easy to determine the rational strategy for the player who makes the second to last move, etc. Backward induction determines that the best move for the investor is to never invest in the trustee and for the trustee to never return if invested in. Given this, according to game theory, a trusting relationship between both members of the dyad can never be formed (Isoni and Sugden, 2019). In contrast, if considering the optimal and most mutually beneficial way to play the game, the investor should always invest their entire endowment, and the trustee should in turn return half of their earnings to the investor. If trust and reciprocation is maintained throughout the task, then both members of the dyad are optimally benefiting from the relationship.

Interestingly, empirical data show that neither of these principles are followed when the Trust Game is played (King-Casas et al., 2005, 2008; Belli et al., 2012; Hula et al., 2021), making it difficult to characterize what leads to fruitful cooperation. Instead, it seems that individuals in the dyad most often lie somewhere closer to the center of the continuum between rational and optimal decision-making. Often in this task, individuals are gaining trust in the beginning of the game; however, as the task nears its end, players tend to invest and return less as it becomes less beneficial for them to continue cooperating (Hula et al., 2021).

Given the importance of cooperative behaviors in the maintenance of social relationships, it is essential to understand the reasons why some adolescents are more willing than others to cooperate with peers. Several factors could predict cooperation, including level of impulsivity (Ibáñez et al., 2016), social abilities (Glaeser et al., 2000; Lee et al., 2015), theory of mind (Di Dio et al., 2020), and social interdependence (Wagner, 1995). Impulsivity – a personality trait associated with lack of prospection, desire for immediate gratification, and risk-taking (Ibáñez et al., 2016) – can make cooperation challenging, as preference for immediate reward hinders reciprocation (Rilling et al., 2002); however, lack of prospection and risk-taking can aid in forming trusting relationships, as people may initially be more trusting in the hopes of acquiring more rewards (Ibáñez et al., 2016). Given this, depending on the facet of impulsivity at play, more impulsive individuals may be better or worse at forming trusting social relationships. Social skills are a complex set of verbal and nonverbal skills used to express and regulate one’s emotions, understand group norms, and interpret the emotions of others (Lee et al., 2015). Unsurprisingly, Lee et al. (2015) found that better social skills were associated with improved cooperation and collaboration and reduced inter- and intra-group conflict; therefore, better social skills should be associated with forming more cooperative relationships. Theory of mind – understanding that another person may perceive and develop feelings toward a situation differently from one’s own and using such information to rationalize and predict behavior – makes cooperation more multifaceted, as it is easier to cooperate with someone whose actions are perceived as being well-intentioned (Di Dio et al., 2020). As such, possessing theory of mind should result in more cooperative behaviors. Finally, social interdependence – how dependent an outcome is on one’s own actions versus those of a group – can greatly affect cooperative behaviors, as individuals tend to cooperate better when the outcome is viewed holistically, rather than attending to each individual’s contribution (Wagner, 1995); therefore, if the individual’s goal is the betterment of the group, they are more likely to act in a manner that promotes cooperation. Another predictor that is likely to influence cooperative behaviors is rejection sensitivity: the tendency to react defensively and jump to negative assumptions in the face of perceived social rejection (Downey et al., 1998). In such situations, greater sensitivity to rejection makes cooperation challenging, as these individuals exhibit a tendency to overreact to simple misunderstandings; moreover, this behavior alienates peers and results in heightened reports of loneliness in these individuals (Downey et al., 1998).

The current study intends to capture the dynamic nature of cooperation among adolescents. To do so, dyads of anonymous peers played an iterative trust game. We examined two hypotheses. The first posits that each player adapts and responds to the other player’s behavior throughout the game. More specifically, we predict that investors will invest increasing amounts of money if the trustee returns at least their initial investment, and failure to do so will result in loss of trust (i.e., little or no investment from the investor). In order to examine how different personality traits influence behavior related to trust and reciprocity – as well as the overall success of the cooperative relationship – several interindividual differences were studied: impulsivity, social skills, social interdependence, and rejection sensitivity. We predict that these interindividual differences will impact the dyadic relationship in the abovementioned ways.

## 2 Method

### 2.1 Participants

Two-hundred and fifty-seven participants in Grades nine through 11 were recruited from 11 classrooms in five schools composed of students from diverse socioeconomic backgrounds and located either in the greater Montreal area or in more rural regions. Nine participants were removed, as they did not play against a peer due to an odd number of students in the classroom, leaving 248 participants [Males = 110, *M*_Age_ = 15.1 years (SD = 0.50)]. Power analysis revealed the sample size provided >80% power to detect a medium effect size in regression analyses. As compensation for their participation, participants received a subset of their earnings from this task corresponding to an amount varying from $0 to $12.

### 2.2 Procedure

Before commencing the study, participants and their parents/guardians provided written consent, in agreement with the guidelines established by Université du Québec à Montréal. Following consent, the participants completed multiple questionnaires, followed by a paper version of the Trust Game approximately 2 weeks later. Prior to beginning the game, the experimenters ensured that the participants understood the task. To achieve this, two examples were demonstrated to all students: (1) showing a fruitful exchange where the investor invested all of their endowment and the trustee returned half, and (2) displayed a futile exchange, where the investor gave nothing to the trustee and the trustee returned nothing.

### 2.3 Measures

#### 2.3.1 Questionnaires

Participants completed four scales assessing different aspects of their personalities: Barratt Impulsiveness Scale, version 11 (BIS-11; Patton et al., 1995; Cronbach’s *α* = 0.79–0.83; 30 items, each scored 1–4); Social Skills Improvement System – Rating Scales (SSIS-RS; Gresham et al., 2011; *α* = 0.75–0.97; 62 items, each scored 1–4); Social Interdependence Scales (SIS; Johnson and Norem-Hebeisen, 1979; α = 0.84–0.88; 22 items, each scored 1–5); and a short 18 items version of the Children’s Rejection Sensitivity Questionnaire, which initially comprised 36 items (CRSQ; Downey et al., 1998; *α* = 0.72–0.84; each item was scored 1–6). The BIS-11 assesses impulsivity levels, with higher scores indicating augmented levels of impulsivity. The SSIS-RS evaluates different constructs related to social skills, including cooperation, responsibility, self-control, assertion, communication, empathy, engagement, and behavior problems. The SIS is composed of three subscales examining an individual’s preference toward cooperative behavior, competitive behavior, and working alone.

#### 2.3.2 Trust game

**Figure 1:**
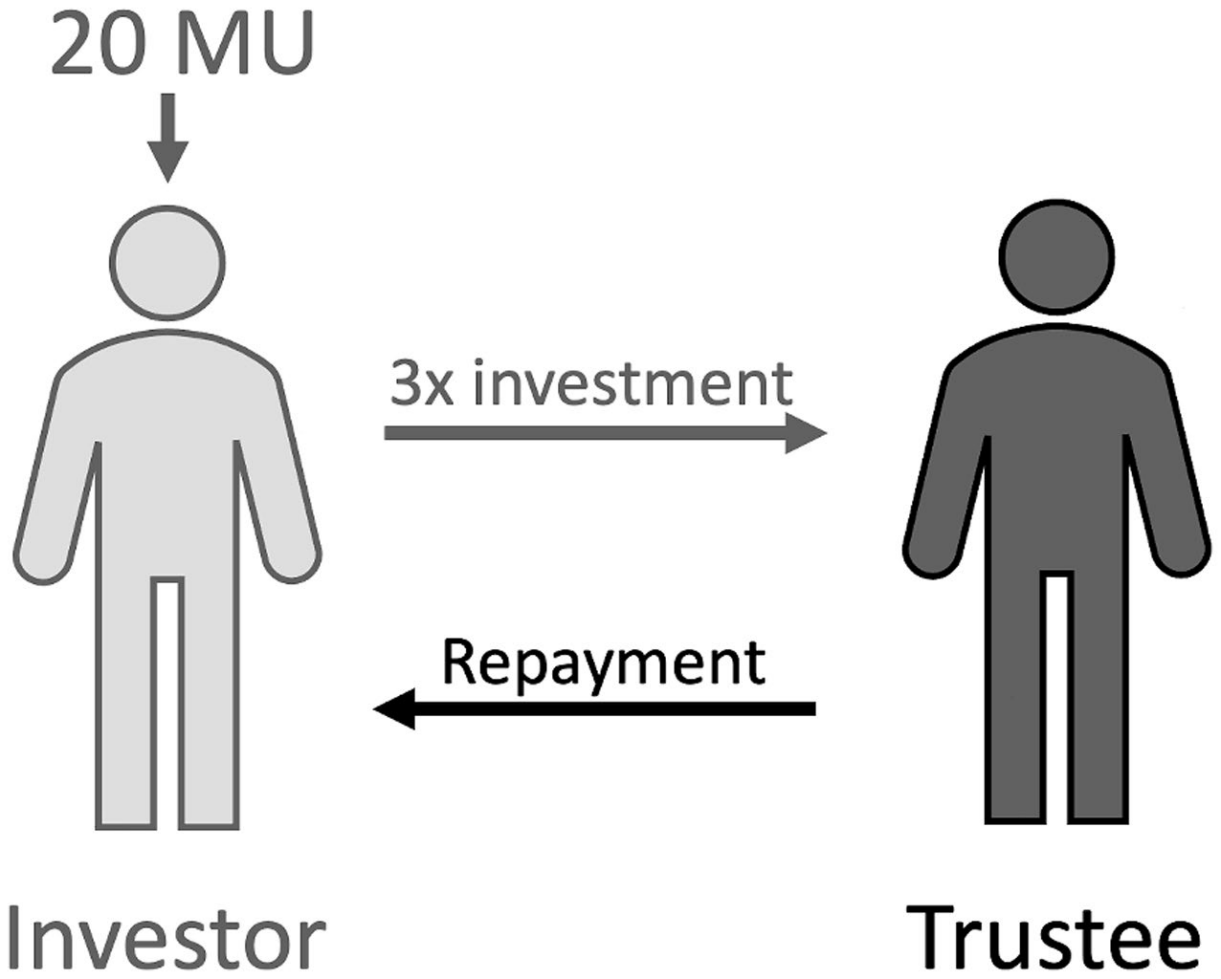
One trial in the trust game paradigm. The investor **(left)** is endowed with 20 monetary units (MU) and gives a portion to the trustee **(right)**. This portion is tripled before being given to the trustee, who then decides how much to repay the investor.

Participants played 10 trials of an iterative Trust Game (adapted from Berg et al., 1995) against another player. Participants were paired randomly with an anonymous classmate. One player in each dyad took the role of the *investor*, and the other, the *trustee*. The investor was endowed with 20 monetary units (MU) each trial and decided how much of this endowment to give to the trustee and how much to keep for themselves (see Figure 1). The amount given by the investor was tripled and given to the trustee. The trustee then decided how much of the investment to keep and how much to return to the investor. Each trial commenced with 20 MU and the investor deciding how much of the endowment to give to the trustee and ends with the trustee returning a fraction (or none) of the investment to the investor. After each round, a research assistant gathered the players’ game sheets, shuffled them, and handed them to the game partner. A complex numerical code was used to associate the dyads of players while maintaining the anonymity of the players. In doing so, the only interaction between the players in the dyad was the amount invested and returned, and the same partners were maintained across all trials. The goal of the game is for each individual to make as much money as possible. Participants were informed that they would earn a percentage of the money made in the task. Importantly, despite both members of the dyad being peers in the same classroom, no student was able to correctly identify their partner when asked at the end of the study.

### 2.4 Data analyses

Using Python 3 (Van Rossum and Drake, 2009), we first examined the pattern of investment and return data across the trials. The mean investment per trial was computed across all investors. In order to make returns more interpretable, percent returns were calculated by dividing the amount returned by the total received by the trustee in said trial. This number was then multiplied by 100, and means were computed per trial across all trustees. Ninety-five percent confidence intervals were calculated for all means. To determine the relationship between the amount invested and earnings for the dyad, total investments across all trials were summed as well as the total earnings for the dyad. A Pearson’s correlation was then performed. The same procedure was performed to determine the correlation between defection number and total earnings for the dyad.

Following this, an exploratory cross-correlation matrix (see Supplementary Table S1) was created in order to determine what variables should be focused on. Next, multilevel models were created using the RStudio software for statistical analysis (RStudio Team, 2020, version 1.3.1056) for each variable of interest to examine their influence on the amount invested by the investor or returned by the trustee on each trial. The predictor variables of interest include impulsivity, social skills, preference for cooperation, competitiveness, and individualism, gender, age, and rejection sensitivity. Average scores for each questionnaire were used and all variables were centered prior to being used in the model. Each model predicted the amount invested or percent returned based on the variable of interest, trial number, trial number squared, as well as an interaction between the trial and variable of interest. Due to the natural shape of the game data, trial number and trial number squared were also added as random effects. In order for the model to be deemed significant, the predictor of interest needed to be significant on its own, separate from its interaction with other predictors. The variable needs to have a significant effect on the intercept in order for the variable to be considered significant. The models are constructed following the parsimony principle (Bates et al., 2015), and having an interaction involving a non-significant variable is counter to this idea. The variables of interest were examined for both players in the dyad, as well as how one partner’s variables may influence the other’s behavior. The multilevel models can be represented by equations 1 through 5:

Level 1:

(1) $$Y_{ij}=\beta_{0j}+\beta_{1j}T_{ij}+\beta_{2j}T_{ij}^{2}+Rij$$

Level 2:


(2) $$\beta_{0j}=\gamma_{00}+\gamma_{01}Z_{j}+U_{0j}$$

(3) $$\beta_{1j}=\gamma_{10}+\gamma_{11}Z_{j}+U_{1j}$$

(4) $$\beta_{2j}=\gamma_{20}+\gamma_{21}Z_{j}+U_{2j}$$

(5) $$\begin{array}{l} Yij=\gamma_{00}+\gamma_{01}Z_{j}+\gamma_{10}T_{ij}+\gamma_{20}T_{ij}^{2}+\gamma_{11}T_{ij}Z_{j}\\ \;\;\;\;\;\;\;\;+\gamma_{21}T_{ij}^{2}Z_{j}+U_{0j}+U_{1j}T_{ij}+U_{2j}T_{ij}^{2}+Rij\; \end{array}$$

In the above equations, investments or percent returned on each trial (*Y_ij_*) are predicted by the level-1 variables trial ($\gamma_{10}$) and trial-squared ($\gamma_{20}$), which both have random slopes ($U_{1j},U_{2j}$), and a level-2 variable representing the questionnaire of interest ($\gamma_{01}$), which interacts with both trial ($\gamma_{11}$) and trial-squared ($\gamma_{21}$). Z_j_ is the participants’ score on the questionnaire of interest. This model also contains the fixed component of the intercept ($\gamma_{00}$), a random component which is the intercept slope (*U_0j_*), and the level-1 residual (*R_ij_*). Importantly, following multilevel notion, the *j* represents individuals and *i* denotes trials.

To determine if the investment on proceeding trials was influenced by current trial number, and if this relationship is mediated by the percent returned by the trustee on the current trial, a multilevel mediation analysis was performed. A multilevel mediation analysis was selected, because percent return and investments were measured across 10 trials; therefore, a repeated measures design is the most appropriate way to examine this data. In this model – as in the previous one – each participant and their questionnaires are at level-2, while each trial is level-1. As the task design necessitates an investment to be made by the investor in order for the trustee to decide how much should be returned, trials where no investment was made were excluded from subsequent data analyses.

Following the notation of Bauer et al. (2006), the mediation model can be depicted in the following equations:

(6) $$M_{ij}=d_{M_{j}}+a_{j}T_{ij}+e_{M_{ij}}$$

(7) $$d_{M_{j}}=d_{0}+u_{0j}$$

(8) $$a_{j}=a_{1}+u_{1j}$$

(9) $$Y_{\left(i+1\right)j}=d_{Y_{j}}+b_{j}M_{ij}+c_{j}^{'}T_{ij}+e_{Y_{ij}}$$

(10) $$d_{Y_{j}}=d_{1}+v_{0j}$$

(11) $$b_{j}=b_{1}+v_{1j}$$

(12) $$c\prime_{j}=c\prime_{1}+v_{2j}\;$$

In the equations, the mediator (*M_ij_*) represents the percent returned by the trustee to the investor on the current trial, *d_Mj_* is the intercept for *M_ij_*, *d_Mj_* represents the intercept for the outcome variable, *T_ij_* is the current trial number, *a_j_* is the effect of *T_ij_* on *M_ij_* holding *Y_(i + 1)j_* constant, *b_j_* is the effect of *M_ij_* on *Y_ij_* holding *T_ij_* constant, *c’_j_* is the effect of *T_ij_* on *Y_ij_* holding *M_ij_* constant. The fixed effects in the model are represented by *a_1_*, *b_1_*, *c’_1_*, *d_0_*, and *d_1_*, while the random effects are represented by *u_0j_*, *u_1j_*, *v_0j_*, *v_1j_*, and *v_2j_*. Finally, the level-1 residual errors for *M_ij_* on *Y_ij_* are assumed to be normally distributed and are represented by *e_Mij_* and *e_Yij_*, respectively.

The multilevel mediation was conducted using the RStudio software for statistical analysis (RStudio Team, 2020, version 1.3.1056) and the Multilevel Mediation package (Falk and Vogel, 2024). A multilevel mediation analysis was performed, with the slope for the *a* and *b* paths being allowed to vary randomly. To determine if the model had a significant indirect effect, the Multilevel Mediation package (Falk and Vogel, 2024) was used to obtain bootstrapped distributions (100 replications, resampled at the participant level). The output of this approach gives 95% confidence intervals, which convey a significant indirect – mediating – effect if the interval does not contain zero. Bootstrapped estimates were used for all paths. *T*-scores and *p*-values were obtained from the mediation model.

The indirect effect – how much *T_ij_* influences *Y_ij_* through *M_ij_* – can be computed by multiplying the estimates from the *a* and *b* pathways (*ab*). Using this information, as well as the direct path (*c’*), we are able to determine the total effect (*c*) of *T* on *Y_ij_* using Equation 13.


(13) $$c=c^{\prime }+ab$$

Once the total effect is known, we are able to calculate the proportion (*prop*) of the effect of *T_ij_* on *Y_ij_* that is mediated through *M_ij_* using Equation 14.


(14) $$\;prop=\frac{ab}{c}\;$$

Subsequent analyses were performed to determine if the significant interindividual differences from the multilevel models (i.e., impulsivity and social skills) moderated the mediation model. Each moderator was applied separately on the model, and on each path; however, they each only significantly moderated the *a* path. For parsimony, the models include only the moderated effect on the *a* path. Given these findings, Pearson (*r*) correlations were computed to determine if trustee impulsivity scores or investor social skills resulted in more personal or dyadic earnings.

## 3 Results

**Figure 2:**
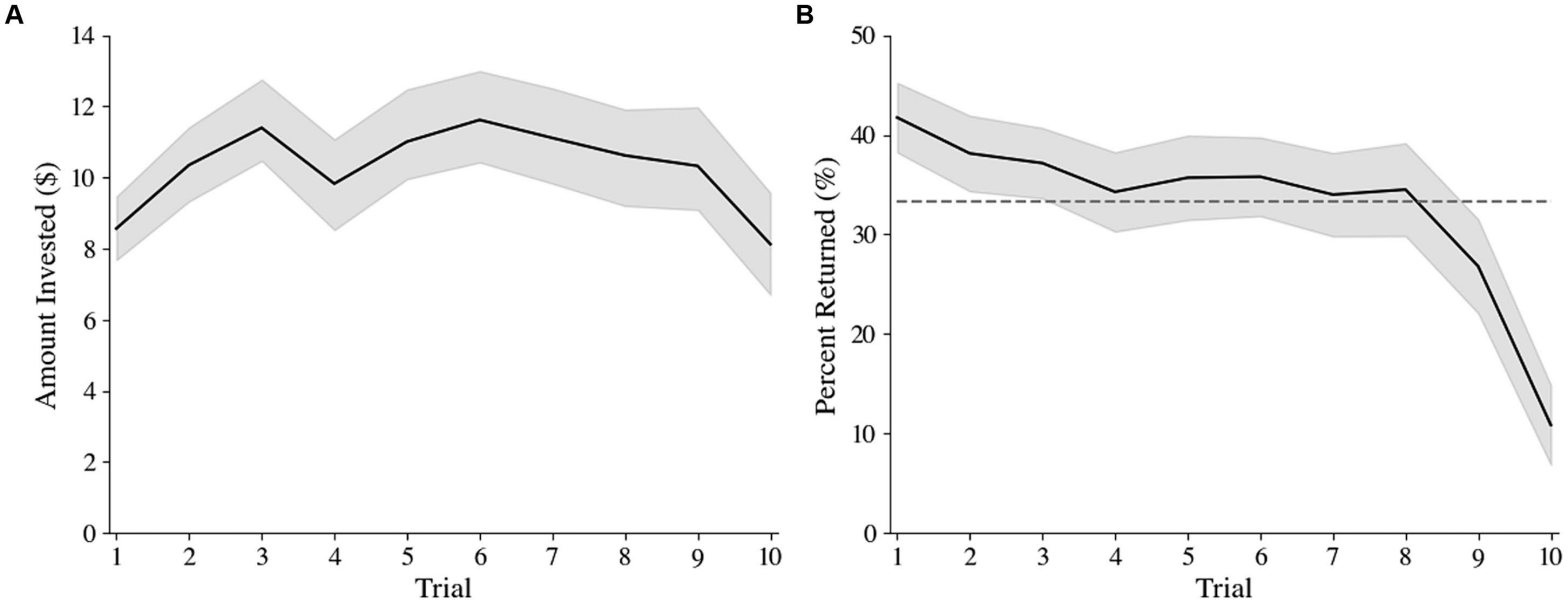
Amount invested by the investor **(A)** and percent returned by the trustee **(B)** each trial. Shaded regions represent 95% confidence intervals. The dotted line represents the point at which the trustee has returned the investor’s initial investment.

To determine the general pattern of investments and returns across trials, the average amount invested by the investor (Figure 2A) and percent returned by the trustee (Figure 2B) were computed per trial. The amount invested has an inverted U shape, where the initial increase in investments represents building of trust within the dyad, and the decrease in the final trial reflects the anticipated defection by the trustee. The decrease in percent returned by the trustee reflects their tendency to defect (i.e., return less than the initial investment, < 33%) in the last few trials. Overall, higher investments lead to higher earnings for the pair (*r* = 0.915, *p* < 0.001), while an increased number of defections results in less dyadic earnings (*r* = −0.265, *p* = 0.003).

**Figure 3:**
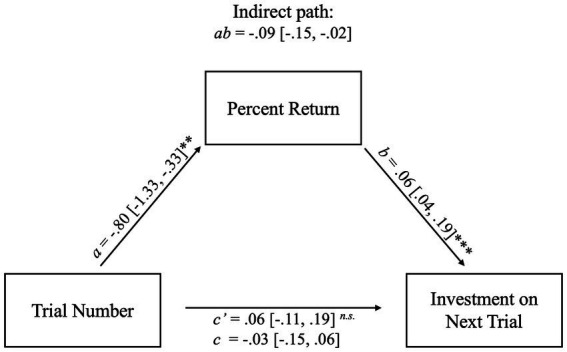
Path diagram for the relationship between trial number and investment on the next trial as mediated by percent return. Mediation of trial number on investment on the next trial through percent return. Percent returned by the *trustee* decreases across trials (*a* path), but greater percent return is associated with greater investments by the *investor* on the next trial (*b* path). There was an indirect effect of trial number on investment in the following trial through percent return (*ab* path). That is, when percent return is greater, the investment on the subsequent trial also increases; however, the percent return decreases across trials which suggests a tendency for *trustees* to defect as the task reaches its end. The indirect path (*ab*) and its confidence intervals were computed using bootstrapped estimates; therefore, no *p-*value was computed. *p < 0.05, **p < 0.01, ***p < 0.001.

Using a multilevel mediation analysis, we observed that the relationship between current trial number and investment on the next trial (*c* path) was mediated by the percent returned by the trustee on the current trial. The total effect of trial number on future investments (*c* path) was found to be −0.03 (95% CI = [−0.15, 0.06]), with the indirect effect comprising 46.0% of the total effect. Using the bootstrapped distributions, the indirect effect was found to have a significant negative influence on investment (*ab* = −0.09 [−0.15, −0.02]). As shown in Figure 3, the *a* path - the influence of trial number on percent return - was statistically significant (*a* = −0.80 [−1.33, −0.33], *t*(1862) = −3.17, *p* = 0.002), as was the *b* path between percent return and investment on the next trial (*b* = 0.06 [0.04, 0.19], *t*(1862) = 6.58, *p* < 0.001). The direct effect of trial number on investments was non-significant (*c’* = −0.06 [−0.11, 0.19], *t*(1862) = 0.89, *p* = 0.37).

Overall, in order to maximize the dyadic earnings, investments need to rise quickly and be maintained. Importantly, this is controlled by the trustee through how much they return to the investor.

**Figure 4:**
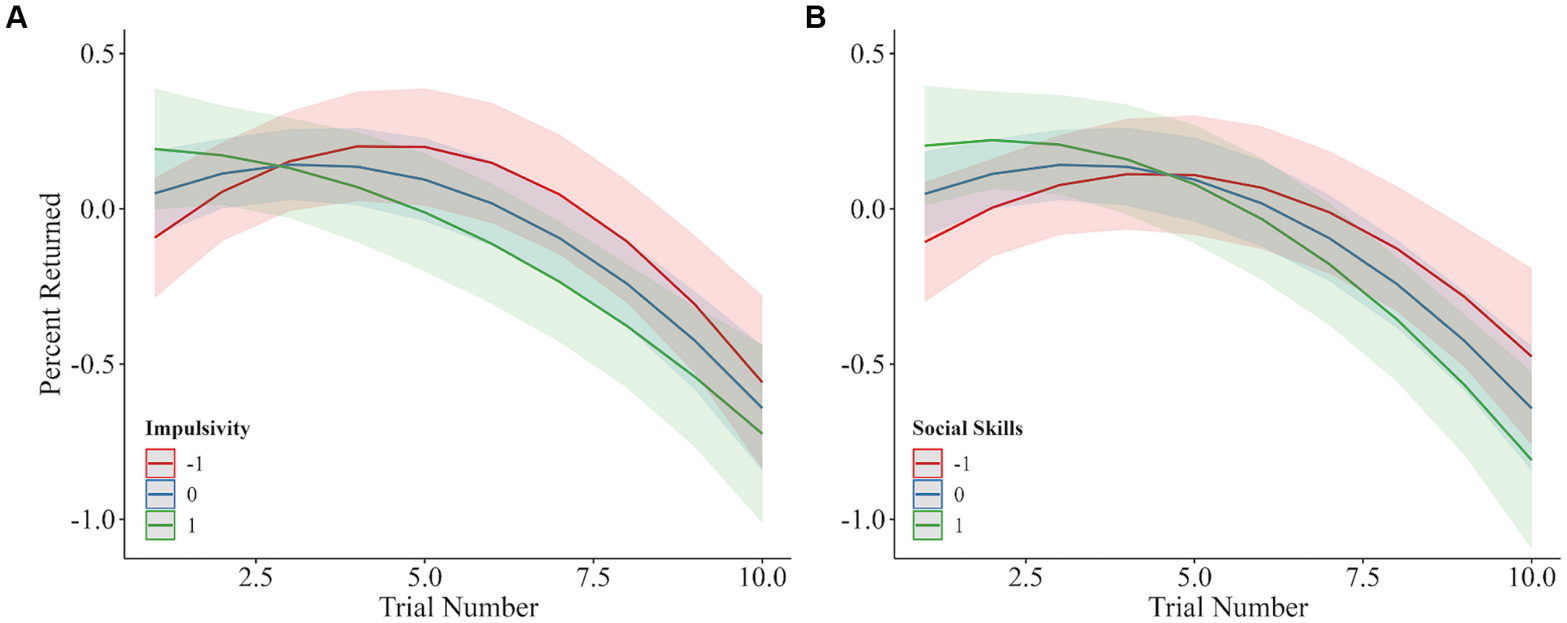
Multilevel model predictions for the percent returned per trial based on the trustee’s impulsivity score **(A)** and the investor’s social skills **(B)**. Shaded regions represent 95% confidence intervals, all values are standardized, and standard deviations are shown.

To provide a complete model of how the variables of interest are influencing the behavior of the players, we examined how each of the variables of interest affected the amount invested or returned. The models show how one’s own personality and personal characteristics influence the amount invested by the investor or the percent returned by the trustee. Since the amount invested and returned (Level 1) were nested within the variables of interest (Level 2), multilevel models were used to account for the dependency of multiple investments and returns. Investments or returns were predicted separately for each variable of interest, with trial number as the variable on the x-axis. Trial number ($\gamma_{10}\;$= 0.08 [0.00, 0.15], *t*(946) = 2.22, *p* = 0.027) was associated with an increase in percent returned by the trustee, while trial number*^2^* ($\gamma_{20}\;$= −0.02 [−0.03, −0.00], *t*(946) = −4.26, *p* < 0.001) was associated with decreased return. Higher trustee impulsivity scores were associated with increased percent return ($\gamma_{01}$ = 0.14 [0.00, 0.28], *t*(122) = 2.04, *p* = 0.044; see Figure 4A), while holding trial number constant. Interestingly, despite the negative interaction between trial number and impulsivity ($\gamma_{11}$ = −0.09 [−0.16, −0.02], *t*(946) = −2.51, *p* = 0.012), there was no interaction between impulsivity and trial number*^2^* ($\gamma_{21}$ = 0.01 [−0.00, 0.02], *t*(946) = 1.81, *p* = 0.07), suggesting that those with higher levels of impulsivity are decreasing their investments across time linearly. For the investors, trial number ($\gamma_{10}$ = 0.08 [0.01, 0.16], *t*(946) = 2.20, *p* = 0.028) and social skills ($\gamma_{01}$ = 0.16 [0.02, 0.29], *t*(122) = 2.23, *p* = 0.027) were both associated with increased percent returned by the trustee, while trial number*^2^* was related to decreased return ($\gamma_{20}$ = −0.02 [−0.03, −0.01], *t*(946) = −4.24, *p* < 0.001; see Figure 4B). There was no significant interaction between trial number and social skills ($\gamma_{11}$ = −0.05 [−0.12, 0.02], *t*(946) = −1.29, *p* = 0.198) or trial number*^2^* and social skills ($\gamma_{21}$ = 0.00 [−0.01, 0.01], *t*(946) = 0.03, *p* = 0.746). See Table 1 for random effects estimates from both models. All other multilevel models were non-significant as the personality predictors did not significantly impact the model’s intercepts (see Tables 2–5). Note, the goals of the models were to investigate the role of personality as a level 2 variable, and the first three rows in the tables reflect level 1 effects, regardless of personality. There was one personality trait – preference for working alone – which had an influence on the slope of trial; however, it did not predict the intercept. For the sake of model parsimony, we do not consider preference for working alone to be a significant predictor in the model. From these null results, we cannot conclude that no effect exists, as such we are unable to draw conclusions based on these findings..

**Table 1:** Estimates from the multilevel models for amount returned based on trustee’s own impulsivity (imp) and investor’s social skills (SS).

	Percent returned (Imp)			Percent returned (SS)		
*Predictors*	*Estimates*	*95% CI*	*p*	*Estimates*	*95% CI*	*p*
(Intercept)	0.050	[−0.088, 0.188]	0.478	0.048	[−0.089, 0.185]	0.492
Trial	0.081	[0.001, 0.153]	0.027	0.082	[0.001, 0.155]	0.028
Trial^2^	−0.018	[−0.026, −0.001]	<0.001	−0.018	[−0.026, −0.001]	<0.001
Imp	0.143	[0.004, 0.281]	0.044			
Imp * Trial	−0.092	[−0.163, −0.020]	0.012			
Imp * Trial^2^	0.007	[−0.001, 0.015]	0.070			
SS				0.155	[0.018, 0.293]	0.027
SS * Trial				−0.048	[−0.121, 0.025]	0.198
SS * Trial^2^				0.001	[−0.007, 0.010]	0.746
*Random effects*						
σ^2^	0.747			0.747		
τ_0_^2^	0.497			0.489		
τ_1_^2^	0.240			0.251		
τ_2_^2^	0.027			0.028		
ICC_adj_	0.423			0.427		
*R* ^2^	0.064, 0.460			0.064, 0.464		

SS, social skills; Imp, impulsivity; ICC_adj_, adjusted ICC; *R*^2^, *R*^2^ marginal, conditional.

**Table 2:** Estimates from the multilevel models for percent returned based on trustee’s personality and characteristics (X).

	Percent returned: impulsivity		Percent returned: social skills		Percent returned: cooperation		Percent returned: competitiveness		Percent returned: preference for working alone		Percent returned: rejection sensitivity		Percent returned: sex		Percent returned: age	
Predictors	Estimates	CI	Estimates	CI	Estimates	CI	Estimates	CI	Estimates	CI	Estimates	CI	Estimates	CI	Estimates	CI
Intercept	0.050	[−0.088, 0.188]	0.047	[−0.093, 0.187]	0.050	[−0.092, 0.188]	0.049	[−0.091, 0.188]	0.049	[−0.091, 0.188]	0.049	[−0.091, 0.189]	0.301	[−0.168, 0.770]	0.043	[−0.099, 0.185]
Trial	0.081*	[0.001, 0.153]	0.083*	[0.001, 0.157]	0.082*	[0.001, 0.156]	0.082*	[0.009, 0.154]	0.082*	[0.009, 0.156]	0.082*	[0.008, 0.155]	0.241	[−0.033, 0.485]	0.089*	[0.014, 0.164]
Trial^2^	−0.018***	[−0.026, −0.010]	−0.018***	[−0.018, −0.010]	−0.018***	[−0.026, −0.010]	−0.018***	[−0.026, −0.010]	−0.018***	[−0.026, −0.010]	−0.018***	[−0.026, −0.010]	−0.041**	[−0.068, −0.015]	−0.018***	[−0.027, −0.010]
*X*	0.143	[0.004, 0.281]	−0.019	[−0.162, 0.125]	−0.028	[−0.169, 0.114]	−0.040	[−0.018, 0.100]	−0.050	[−0.189, 0.009]	−0.001	[−0.142, 0.140]	−0.160	[−0.442, 0.125]	−0.074	[−0.218, 0.070]
*X* * Trial	−0.092*	[−0.163, −0.020]	0.029	[−0.046, 0.103]	−0.004	[−0.077, 0.070]	0.063	[−0.009, 0.134]	0.006	[−0.066, 0.079]	−0.029	[−0.103, 0.044]	−0.100	[−0.248, 0.047]	0.012	[−0.062, 0.087]
*X* * Trial^2^	0.007	[−0.001, 0.015]	−0.001	[−0.001, 0.007]	0.000	[−0.008, 0.008]	−0.008	[−0.016, 0.000]	0.001	[−0.008, 0.008]	0.003	[−0.006, 0.011]	0.015	[−0.001, 0.031]	−0.002	[−0.010, 0.006]
*Random effects*																
σ^2^	0.747		0.747		0.747		0.747		0.747		0.747		0.747		0.747	
τ_0_^2^	0.497		0.516		0.514		0.512		0.512		0.515		0.508		0.520	
τ_1_^2^	0.240		0.256		0.257		0.246		0.257		0.255		0.250		0.259	
τ_2_^2^	0.027		0.028		0.028		0.026		0.028		0.028		0.027		0.028	
ICC_adj_	0.423		0.423		0.423		0.428		0.432		0.430		0.424		0.435	
R^2^	0.064, 0.460		0.062, 0.461		0.056, 0.462		0.058, 0.462		0.055, 0.463		0.057, 0.463		0.068, 0.463		0.058, 0.467	

CI, 95% confidence intervals; X, predictor of interest; ICC_adj_, adjusted ICC; *R*^2^, *R*^2^ marginal, conditional *p < 0.05, **p < 0.01, ***p < 0.001.

**Table 3:** Estimates from the multilevel models for amount invested based on investor’s personality and characteristics (X).

	Investment: impulsivity		Investment: social skills		Investment: cooperation		Investment: competitiveness		Investment: preference for working alone		Investment: rejection sensitivity		Investment: sex		Investment: age	
Predictors	Estimates	CI	Estimates	CI	Estimates	CI	Estimates	CI	Estimates	CI	Estimates	CI	Estimates	CI	Estimates	CI
Intercept	−0.206**	[−0.325, −0.087]	−0.206**	[−0.325, −0.087]	−0.206**	[−0.325, −0.086]	−0.206**	[−0.324, −0.087]	−0.206**	[−0.325, −0.087]	−0.206**	[−0.325, −0.086]	−0.131	[−0.517, 0.254]	−0.185**	[−0.304, −0.065]
Trial	0.161***	[0.096, 0.227]	0.161***	[0.095, 0.227]	0.161***	[0.096, 0.227]	0.161***	[0.096, 0.227]	0.161***	[0.097, 0.225]	0.161***	[0.096, 0.227]	0.234*	[0.022, 0.446]	0.165***	[0.099, 0.232]
Trial^2^	−0.018***	[−0.025, −0.011]	−0.018***	[−0.025, −0.011]	−0.018***	[−0.025, −0.011]	−0.018***	[−0.025, −0.011]	−0.018***	[−0.025, −0.011]	−0.018***	[−0.025, −0.011]	−0.025*	[−0.047, −0.002]	−0.019***	[−0.026, −0.012]
*X*	−0.047	[−0.167, 0.074]	0.057	[−0.064, 0.177]	−0.005	[−0.126, 0.116]	0.086	[−0.034, 0.206]	0.068	[−0.052, 0.188]	−0.043	[−0.163, 0.078]	−0.049	[−0.290, 0.193]	−0.070	[−0.190, 0.051]
*X* * Trial	−0.028	[−0.094, 0.038]	0.006	[−0.060, 0.072]	−0.046	[−0.111, 0.020]	0.024	[−0.042, 0.090]	0.080*	[0.015, 0.144]	−0.010	[−0.076, 0.056]	−0.048	[−0.179, 0.084]	−0.024	[−0.091, 0.042]
*X* * Trial^2^	0.003	[−0.004, 0.010]	−0.001	[−0.007, 0.007]	0.006	[−0.001, 0.013]	−0.003	[−0.010, 0.004]	−0.008*	[−0.015, 0.001]	0.001	[−0.006, 0.008]	0.004	[−0.001, 0.018]	0.001	[−0.006, 0.008]
*Random effects*																
σ^2^	0.677		0.677		0.677		0.677		0.677		0.677		0.677		0.666	
τ_0_^2^	0.417		0.415		0.419		0.411		0.414		0.417		0.419		0.401	
τ_1_^2^	0.250		0.252		0.247		0.251		0.239		0.252		0.251		0.259	
τ_2_^2^	0.026		0.026		0.026		0.026		0.025		0.026		0.026		0.026	
ICC_adj_	0.526		0.528		0.527		0.526		0.560		0.529		0.528		0.534	
R^2^	0.028, 0.539		0.025, 0.539		0.022, 0.539		0.029, 0.540		0.060, 0.539		0.021, 0.539		0.023, 0.539		0.045, 0.555	

CI, 95% confidence intervals; X, predictor of interest; ICC_adj_, adjusted ICC; *R*^2^, *R*^2^ marginal, conditional *p < 0.05, **p < 0.01, ***p < 0.001.

**Table 4:** Estimates from the multilevel models for amount invested based on trustee’s personality and characteristics (X).

	Investment: impulsivity		Investment: social skills		Investment: cooperation		Investment: competitiveness		Investment: preference for working alone		Investment: rejection sensitivity		Investment: sex		Investment: age	
Predictors	Estimates	CI	Estimates	CI	Estimates	CI	Estimates	CI	Estimates	CI	Estimates	CI	Estimates	CI	Estimates	CI
Intercept	−0.206**	[−0.323, −0.088]	−0.206**	[−0.325, −0.086]	−0.206**	[−0.325, −0.086]	−0.206**	[−0.325, −0.087]	−0.206**	[−0.325, −0.087]	−0.206**	[−0.325, −0.086]	−0.148	[−0.549, 0.253]	−0.211**	[−0.333, −0.089]
Trial	0.161***	[0.096, 0.227]	0.161***	[0.096, 0.227]	0.161***	[0.096, 0.227]	0.161***	[0.096, 0.227]	0.161***	[0.096, 0.227]	0.161***	[0.096, 0.227]	0.266*	[0.046, 0.486]	0.166***	[0.100, 0.232]
Trial^2^	−0.018***	[−0.025, −0.011]	−0.018***	[−0.025, −0.011]	−0.018***	[−0.025, −0.011]	−0.018***	[−0.025, −0.011]	−0.018***	[−0.025, −0.011]	−0.018***	[−0.025, −0.011]	−0.025*	[−0.048, −0.002]	−0.019***	[−0.026, −0.012]
*X*	0.115	[−0.004, 0.234]	−0.034	[−0.154, 0.087]	−0.028	[−0.148, 0.092]	−0.052	[−0.172, 0.069]	0.042	[−0.078, 0.163]	−0.022	[−0.142, 0.099]	−0.036	[−0.281, 0.208]	−0.048	[−0.171, 0.075]
*X* * Trial	−0.033	[−0.098, 0.033]	−0.032	[−0.097, 0.034]	−0.011	[−0.077, 0.055]	0.031	[−0.035, 0.096]	0.015	[−0.051, 0.080]	−0.044	[−0.109, 0.021]	−0.066	[−0.199, 0.066]	−0.057	[−0.123, 0.009]
*X* * Trial^2^	0.002	[−0.005, 0.009]	0.006	[−0.001, 0.013]	0.001	[−0.007, 0.008]	−0.004	[−0.011, 0.003]	−0.003	[−0.010, 0.004]	0.005	[−0.002, 0.012]	0.004	[−0.010, 0.018]	0.005	[−0.002, 0.012]
*Random effects*																
σ^2^	0.677		0.677		0.677		0.677		0.677		0.677		0.677		0.670	
τ_0_^2^	0.403		0.418		0.418		0.416		0.418		0.419		0.419		0.435	
τ_1_^2^	0.250		0.250		0.251		0.250		0.251		0.250		0.250		0.251	
τ_2_^2^	0.026		0.026		0.026		0.026		0.026		0.026		0.026		0.026	
ICC_adj_	0.530		0.528		0.529		0.530		0.530		0.527		0.525		0.528	
*R* ^2^	0.020, 0.540		0.023, 0.539		0.022, 0.539		0.019, 0.539		0.020, 0.540		0.025, 0.539		0.031, 0.540		0.044, 0.549	

CI, 95% confidence intervals; X, predictor of interest; ICC_adj_, Adjusted ICC; *R*^2^, *R*^2^ marginal, conditional *p < 0.05, **p < 0.01, ***p < 0.001.

**Table 5:** Estimates from the multilevel models for percent returned based on investor’s personality and characteristics (X).

	Percent returned: impulsivity		Percent returned: social skills		Percent returned: cooperation		Percent returned: competitiveness		Percent returned: preference for working alone		Percent returned: rejection sensitivity		Percent returned: sex		Percent returned: age	
Predictors	Estimates	CI	Estimates	CI	Estimates	CI	Estimates	CI	Estimates	CI	Estimates	CI	Estimates	CI	Estimates	CI
Intercept	0.049	[−0.090, 0.187]	0.048	[−0.089, 0.185]	0.049	[−0.091, 0.189]	0.048	[−0.091, 0.188]	0.049	[−0.091, 0.189]	0.048	[−0.091, 0.189]	−0.339	[−0.789, 0.110]	0.051	[−0.087, 0.188]
Trial	0.082*	[0.008, 0.155]	0.082*	[0.009, 0.155]	0.081*	[0.008, 0.155]	0.082*	[0.009, 0.156]	0.081*	[0.007, 0.154]	0.082*	[0.009, 0.155]	0.188	[−0.051, 0.428]	0.083*	[0.008, 0.158]
Trial^2^	−0.018***	[−0.026, −0.010]	−0.018***	[−0.018, −0.010]	−0.018***	[−0.026, −0.010]	−0.018***	[−0.026, −0.010]	−0.018***	[−0.026, −0.010]	−0.018***	[−0.026, −0.010]	−0.024	[−0.051, 0.002]	−0.018***	[−0.027, −0.010]
*X*	−0.126	[−0.265, 0.013]	0.155*	[0.018, 0.293]	−0.037	[−0.177, 0.103]	0.058	[−0.083, 0.199]	0.025	[−0.116, 0.165]	0.055	[−0.085, 0.195]	0.252	[−0.028, 0.532]	−0.026	[−0.166, 0.113]
*X* * Trial	0.028	[−0.046, 0.102]	−0.048	[−0.121, 0.025]	−0.017	[−0.091, 0.057]	−0.004	[−0.078, 0.071]	0.043	[−0.031, 0.117]	−0.068	[−0.140, 0.004]	−0.069	[−0.216, 0.078]	−0.027	[−0.104, 0.049]
*X* * Trial^2^	−0.003	[−0.011, 0.005]	0.001	[−0.007, 0.010]	0.001	[−0.007, 0.009]	−0.001	[−0.009, 0.007]	−0.005	[−0.014, 0.003]	0.007	[−0.001, 0.015]	0.004	[−0.012, 0.021]	0.002	[−0.007, 0.011]
*Random effects*																
σ^2^	0.747		0.747		0.747		0.747		0.747		0.747		0.747		0.727	
τ_0_^2^	0.500		0.490		0.513		0.511		0.516		0.511		0.501		0.488	
τ_1_^2^	0.255		0.251		0.256		0.256		0.254		0.248		0.254		0.264	
τ_2_^2^	0.028		0.028		0.028		0.028		0.027		0.027		0.028		0.029	
ICC_adj_	0.423		0.423		0.423		0.431		0.432		0.428		0.430		0.447	
R^2^	0.061, 0.462		0.064, 0.464		0.060, 0.462		0.055, 0.463		0.060, 0.464		0.060, 0.463		0.058, 0.463		0.065, 0.483	

CI, 95% confidence intervals; X, predictor of interest; ICC_adj_, adjusted ICC; *R*^2^, R^2^ marginal, conditional *p < 0.05, **p < 0.01, ***p < 0.001.

**Figure 5:**
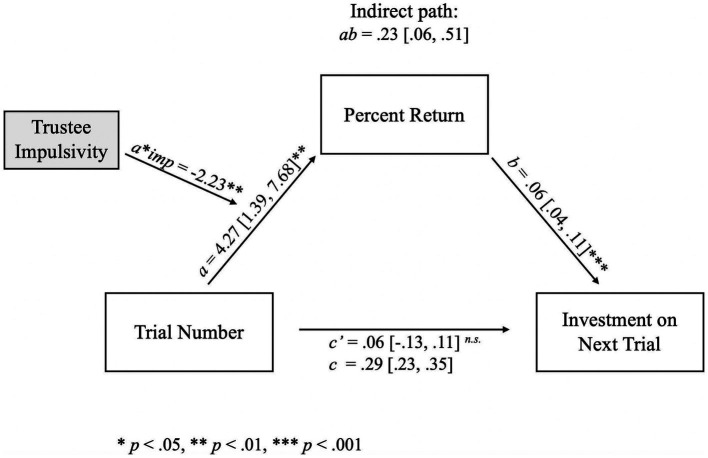
Path diagram for the relationship between trial number and investment on the next trial as mediated by percent return and moderated by trustee impulsivity. Mediation of trial number on investment on the next trial through percent return. Percent returned by the *trustee* increases across trials (*a* path), but impulsivity lessened the returns (a*imp path). Greater percent return is associated with greater investments by the *investor* on the next trial (*b* path). There was an indirect effect of trial number on investment in the following trial through percent return (*ab* path). That is, when percent return is greater, the investment on the subsequent trial also increases; however, the percent return decreases across trials when impulsivity is higher, which suggests a tendency for more impulsive *trustees* to defect as the task reaches its end. Impulsivity did not moderate any other paths in the model, implying that *trustee* impulsivity only affects percent return. The indirect path (*ab*) and its confidence intervals were computed using bootstrapped estimates; therefore, no *p-*value was computed. *p < 0.05, **p < 0.01, ***p < 0.001.

To explore the influence of both impulsivity and social skills on cooperative behavior, each personality trait was separately added as a moderator on to the multilevel mediation model. When impulsivity was added as a moderator, the relationship between current trial number and investment on the next trial (*c* path) was mediated by the percent returned by the trustee on the current trial. The total effect of trial number on future investments (*c* path) was found to be 0.29 [0.23, 0.35], with the indirect effect comprising 78.3% of the total effect. Using the bootstrapped distributions, the indirect effect was found to have a significant positive influence on investment (*ab* = 0.23 [0.06, 0.51]). As shown in Figure 5, the *a* path between trial number and percent return was statistically significant (*a* = 4.27 [1.39, 7.68], *t*(1860) = 2.72, *p* = 0.007), showing that trial number positively impacts the percent returned; moreover, the effect of trial number on percent return was negatively moderated by the trustee’s impulsivity score (a*imp = −2.23, *t*(1860) = −3.27, *p* = 0.001), and the *b* path between percent return and investment on the next trial also had a significant effect (*b* = 0.06 [0.04, 0.11], *t*(1860) = 6.82, *p* < 0.001), such that increasing percent returned on the current trial increased investor investments on the next trial. The direct effect of trial number on investments was non-significant (*c’* = 0.06 [−0.13, 0.11], *t*(1860) = 0.89, *p* = 0.37).

**Figure 6:**
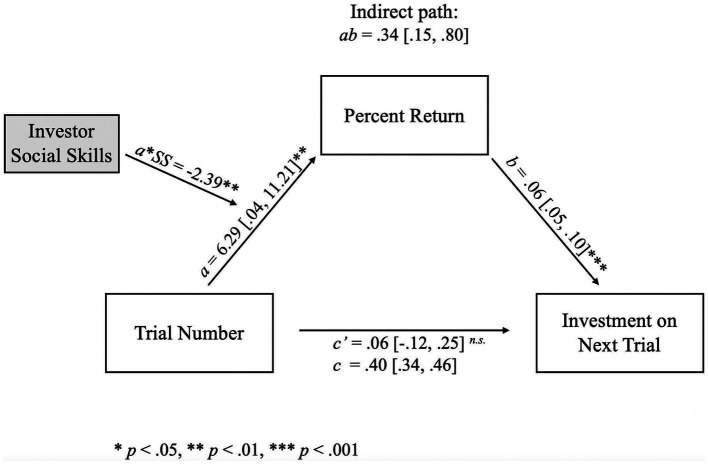
Path diagram for the relationship between trial number and investment on the next trial as mediated by percent return and moderated by investor social skills. Mediation of trial number on investment on the next trial through percent return. Percent returned by the *trustee* increases across trials (*a* path), but *investor* social skills lessened the returns (a*SS path). Greater percent return is associated with greater investments by the *investor* on the next trial (*b* path). There was an indirect effect of trial number on investment in the following trial through percent return (*ab* path). That is, when percent return is greater, the investment on the subsequent trial also increases; however, the percent return decreases across trials when the investor has greater social skills, which suggests a tendency for *trustees* to defect when they know the *investor* will try to maintain their relationship through continued investments. Social skills did not moderate any other paths in the model, implying that *investor* social skills only affect percent return. The indirect path (*ab*) and its confidence intervals were computed using bootstrapped estimates; therefore, no *p-*value was computed. *p < 0.05, **p < 0.01, ***p < 0.001.

When investor social skills was added as a moderator, the relationship between current trial number and investment on the next trial (*c* path) was mediated by the percent returned by the trustee on the current trial. The total effect of trial number on future investments (*c* path) was found to be 0.40 [0.34, 0.46], with the indirect effect comprising 82.9% of the total effect. Using the bootstrapped distributions, the indirect effect was found to have a significant positive influence on investment (*ab* = 0.34 [0.15, 0.80]). As shown in Figure 6, the *a* path between trial number and percent return was statistically significant (*a* = 6.29 [0.04, 11.21], *t*(1860) = 2.66, *p* = 0.008), showing that as trial number increases, the percent returned by the trustee increases accordingly. The relationship between trial number and percent returned was negatively moderated by the investor’s social (a*SS = −2.39, *t*(1860) = −3.02, *p* = 0.003). The *b* path - between percent return and investment on the next trial – also had a significant effect, such that increasing percent returned by the trustee increased the amount invested by the investor on the proceeding trial (*b* = 0.06 [0.05, 0.10], *t*(1860) = 6.70, *p* < 0.001). The direct effect of trial number on investments did not reach significance (*c’* = 0.06 [−0.12, 0.25], *t*(1860) = 0.85, *p* = 0.39).

In order to further examine if impulsivity and social skills are beneficial for cooperative behavior, their association with earnings was assessed. Notably, trustee’s impulsivity was not associated with increased personal (*r* = 0.080, *p* = 0.379) or dyadic earnings (*r* = 0.074, *p* = 0.414), suggesting that this behavior is detrimental to the player. Similarly, investor’s social skills were also not correlated with own earnings (*r* = 0.004, *p* = 0.969) or those of the dyad (*r* = 0.081, *p* = 0.368), indicating that this behavior does not benefit the player.

## 4 Discussion

The study examined how trusting and cooperative relationships were formed and broken throughout the Trust Game. In agreement with our hypotheses, we found that each member of the dyad adaptively altered their behavior in response to the other player. Investments had an inverted “U” shape, where trust increased across trials, until the mid-way point where it began to decrease due to anticipation of defection near the end of the game. We also observed that trial number was negatively associated with investments on the subsequent trial, something that is influenced by the amount of money returned by the trustee. Interestingly, the trustee’s impulsivity score and investor’s social skills each moderated the mediating effect of trial number on investments on the proceeding trial through percent return, despite neither of these traits being associated with greater earnings. The investor’s social skills and trustee’s impulsivity score were negatively associated with returns and reciprocity.

### 4.1 Cooperative behavior: an interactive process

In line with previous literature using an iterative version of the Trust Game in an adolescent sample playing against computers (Hula et al., 2021), and in an adult sample playing against unknown adults (King-Casas et al., 2005), we also found that trust increased across trials for the first half of the game, before decreasing in the second half. These papers also displayed the same pattern of trustee reciprocation as we observed, where reciprocation starts high and decreases across time. Such patterns of data reflect that the dyad is developing a trusting and mutually beneficial relationship with each other, which will be maintained as long as investments continue and returns are higher than the initial investment. Along with Hula and colleagues, we suggest that high initial returns are used by the trustee in order to coax additional investments out of the investor. Despite the similarities between our study and that of King-Casas et al. (2005), our study uses dyads of anonymous adolescent peers to examine how trust develops; moreover, adolescents have been shown to display less trust and reciprocity in the Trust Game than adults (Belli et al., 2012). This key difference allows us to assess cooperative behaviors during an important development period as well as being more realistic as participants are engaging with members of their own peer group. The finding that reciprocity is a strong predictor of trust has also been supported by early work using the Trust Game (Berg et al., 1995). The study used a one-shot version of the task and found that increasing investments resulted in increased returns. Despite Hula et al. (2021) showing that returns decreased across trials, to our knowledge, the present study is the first to find that returns and reciprocity mediate the relationship between trial number and investments on future trials. These show the importance of reciprocity in creating and maintaining trust, even though reciprocity dwindles across time when there is a known end to the relationship. Research by Belli et al. (2012) found that withholding the total number of trials from the participants prevents backward induction from occurring, as they are unable to determine the optimal time to defect.

### 4.2 Association between cooperation and interindividual characteristics

Impulsivity seems like it should hinder relationships and be solely self-benefiting, but that may not be the case. A study performed by Ibáñez et al. (2016) used a modified one-shot binary-option version of the Trust Game, where participants were partnered with real individuals. In accordance with our findings, they also observed that lack of reciprocity by the trustee was associated with impulsivity; although, they observed that high levels of impulsivity resulted in lower returns. Impulsive people – especially adolescents – often display a preference for immediate rewards and lack premeditation (Bari and Robbins, 2013), which can have both positive and negative consequences. If the trustee defects too early – by decreasing returns – the investor may decrease or withhold investments as a result. This leads to less money made by the trustee; however, a patient investor may continue to cooperate despite poor returns. If the trustee defects too late, they also earn less money, because they returned too much to the investor. If the trustee continually returns slightly more than invested, and defects at the correct time, their preference for immediate rewards can lead to more money being earned. Despite this, we did not observe that impulsivity was related to greater earnings overall.

Social skills are important in forming and maintaining trusting cooperative relationships, but being too social may lead to being taken advantage of. Research by Glaeser et al. (2020) investigated how social skills influenced trust and earnings in a modified one-shot version of the Trust Game, where your partner is not anonymous and you may have an existing social relationship. The authors found that greater social skills were associated with greater returns. This implies that either (1) individuals with greater social skills are returning more money, in order to maintain the relationship with their partner, or (2) the partner wants to maintain a good relationship with them, and could potentially benefit from this in the future. These findings contradict our observation that investors’ social skills are associated with decreased return, reflecting less reciprocation. In spite of these players not earning less money as a result of their social skills, they are being taken advantage of by the trustee who may expect continued investments despite lessening returns. The idea is that an individual with better social skills will work harder to maintain the relationship and may give their partner more chances than they should; although, good social skills may also include being suspicious of one’s partner and trying to avoid being cheated out of returns. Since the investor is playing the Trust Game with one other person, it is in their best interest to cooperate, as cooperation leads to the highest individual and dyadic earnings. Indeed, if the investor never invested in the trustee, the amount earned will never be as high as it is during successful cooperation; therefore, it is in their best interest to cooperate, even for someone who feels like their kindness is being exploited.

### 4.3 Limitations and future directions

A few limitations should be noted. The first limitation relates to the ecological validity of the task, in that it is challenging to know how our findings will translate into the real world. Specifically, because the task requires short-lived cooperation it is unclear whether it adequately captures relationships in the outside world, unlike in the game. More trials are needed to better examine the influence of interindividual differences in this type of task. In addition, since the task consists of a very strong social situation, where the benefits of cooperation are clear to all, using a more ambiguous social situation may result in stronger effects mainly related to personality.

Despite these limitations, this study improves upon the existing literature in several ways: (1) real interaction between adolescent peers in the dyad, and (2) the task is iterative. By having real players interact, we are able to obtain much more information than using questionnaires. For instance, knowing that there is an end to cooperation is detrimental to the relationship, as many trustees defect and investors do not invest during the last trial. Having a one-off interaction does not reflect long-term cooperation, which is the goal of many of these studies. It is important to give people the impression that cooperation is long-term. To the best of our knowledge, this is also the first study to show the importance of reciprocity in maintaining relationships between real adolescent players, as lack of reciprocity will lead to a rupture in trust that is evident across trials.

## 5 Conclusion

Taken together, these findings reveal that adolescent cooperation is a complex behavior which relies on trust and reciprocity, and anything that inhibits those hinders the relationship. We have learned that cooperation decreases near the end of the relationship and depends on reciprocity. Importantly, this study shows that cooperation is fluid, and changes following positive and negative interactions. Personality traits – like impulsivity and social skills – can both influence how successful the relationships are. Impulsivity makes it more challenging to form and maintain trusting relationships in the long-term, since the individual is often preoccupied with short-term gains. This is especially true in a task like the Trust Game where cooperation is highly constrained by the task. Even though greater social skills are expected to promote trusting and cooperative relationships, it can also be associated with lack of reciprocation from the partner.

## Data availability statement

The raw data supporting the conclusions of this article will be made available by the authors, without undue reservation.

## Ethics statement

The studies involving humans were approved by the Institutional Review Board at the Université du Québec à Montréal. The studies were conducted in accordance with the local legislation and institutional requirements. Written informed consent for participation in this study was provided by the participants’ legal guardians/next of kin.

## Author contributions

TB: Formal analysis, Software, Validation, Visualization, Writing – original draft, Writing – review & editing. IP: Conceptualization, Data curation, Funding acquisition, Investigation, Methodology, Project administration, Resources, Supervision, Writing – review & editing. MR: Conceptualization, Methodology, Supervision, Writing – review & editing.
