# Supplementary material for: It takes two: examining the dynamic nature of cooperative behavior in adolescents
Source: Front Psychol. 2024 Apr 29;15:1269016. doi: 10.3389/fpsyg.2024.1269016 (PMC11089236; doi:10.3389/fpsyg.2024.1269016)
Supplement: Supplementary file 1 [file Table_1.DOCX]

**Supplementary Materials**

**Supplementary Table S1**

*Correlation coefficients (Pearson’s r) between demographics, personality variables, and task earnings for the Investor (I) and Trustee (T).*

|  | **Sex (*I*)** | **Age (*I*)** | **Coop (*I*)** | **Comp (*I*)** | **WA (*I*)** | **SS (*I*)** | **Imp (*I*)** | **RS (*I*)** | **Sex (*T*)** | **Age (*T*)** | **Coop (*T*)** | **Comp (*T*)** | **WA (*T*)** | **SS (*T*)** | **Imp (*T*)** | **RS (*T*)** | **Total Earned (*I*)** | **Total Earned (*T*)** | **Total Earned (Dyad)** |
| --- | --- | --- | --- | --- | --- | --- | --- | --- | --- | --- | --- | --- | --- | --- | --- | --- | --- | --- | --- |
| **Sex (*I*)** | - |  |  |  |  |  |  |  |  |  |  |  |  |  |  |  |  |  |  |
| **Age (*I*)** | -.17 | - |  |  |  |  |  |  |  |  |  |  |  |  |  |  |  |  |  |
| **Coop (*I*)** | -.07 | .02 | - |  |  |  |  |  |  |  |  |  |  |  |  |  |  |  |  |
| **Comp (*I*)** | -.11 | -.05 | -.07 | - |  |  |  |  |  |  |  |  |  |  |  |  |  |  |  |
| **WA (*I*)** | .24 | -.08 | -.44* | .17 | - |  |  |  |  |  |  |  |  |  |  |  |  |  |  |
| **SS (*I*)** | .11 | .01 | .47* | .11 | -.01 | - |  |  |  |  |  |  |  |  |  |  |  |  |  |
| **Imp (*I*)** | -.07 | .02 | -.08 | -.28* | -.28* | -.39* | - |  |  |  |  |  |  |  |  |  |  |  |  |
| **RS (*I*)** | .18 | .04 | -.10 | .06 | -.03 | -.12 | .08 | - |  |  |  |  |  |  |  |  |  |  |  |
| **Sex (*T*)** | -.04 | .26* | -.04 | -.34* | .04 | -.10 | .10 | .04 | - |  |  |  |  |  |  |  |  |  |  |
| **Age (*T*)** | -.17 | .48* | -.04 | -.15 | -.13 | -.05 | .18 | -.06 | .09 | - |  |  |  |  |  |  |  |  |  |
| **Coop (*T*)** | -.03 | -.02 | .03 | -.27* | -.01 | .07 | .09 | .06 | .07 | .14 | - |  |  |  |  |  |  |  |  |
| **Comp (*T*)** | .11 | -.09 | .01 | .16 | -.02 | -.02 | -.10 | .04 | -.25* | -.13 | -.13 | - |  |  |  |  |  |  |  |
| **WA (*T*)** | -.03 | -.08 | -.20 | .20 | .12 | -.22 | .07 | .08 | .14 | -.13 | -.58* | .12 | - |  |  |  |  |  |  |
| **SS (*T*)** | .11 | .09 | .09 | -.12 | -.19 | .02 | .02 | .08 | .16 | .14 | .23 | .05 | -.10 | - |  |  |  |  |  |
| **Imp (*T*)** | .10 | -.05 | -.14 | .07 | .06 | -.00 | .01 | -.06 | -.20 | -.00 | .03 | -.15 | -.13 | -.52* | - |  |  |  |  |
| **RS (*T*)** | .02 | .05 | .08 | -.03 | -.03 | .08 | .05 | .08 | .14 | -.17 | -.06 | .03 | .05 | -.23* | .17 | - |  |  |  |
| **Total Earned (*I*)** | -.15 | -.09 | -.14 | .18 | .19* | -.01 | -.16 | -.05 | -.14 | -.15 | -.15 | .04 | .02 | .08 | -.07 | -.04* | - |  |  |
| **Total Earned (*T*)** | -.11 | -.21* | -.00 | .08 | .25* | .17 | -.11 | -.09 | -.12 | -.18 | -.04 | -.04 | .06 | -.06 | .08 | -.11* | .21* | - |  |
| **Total Earned (Dyad)** | .052 | -.21* | -.067 | .15 | .29* | .13 | -.16* | -.09 | -.16 | -.21* | -.11 | -.02 | .06 | -.01 | .03 | -.11* | .64* | .89* | - |

*Note:* Coop = cooperative behaviour (SIS), Comp = competitive behaviour (ISS), WA = preference for working alone (SIS), SS = social skills (SSIS-RS), imp = impulsivity (BIS-11), RS = rejection sensitivity (CRSQ) **p* < .05
